# Local shape feature fusion for improved matching, pose estimation and 3D object recognition

**DOI:** 10.1186/s40064-016-1906-1

**Published:** 2016-03-08

**Authors:** Anders G. Buch, Henrik G. Petersen, Norbert Krüger

**Affiliations:** Maersk Mc-Kinney Moller Institute, University of Southern Denmark, Odense, Denmark

**Keywords:** 3D shape descriptors, 3D object recognition, Shape matching, Feature fusion

## Abstract

We provide new insights to the problem of shape feature description and matching, techniques that are often applied within 3D object recognition pipelines. We subject several state of the art features to systematic evaluations based on multiple datasets from different sources in a uniform manner. We have carefully prepared and performed a neutral test on the datasets for which the descriptors have shown good recognition performance. Our results expose an important fallacy of previous results, namely that the performance of the recognition system does not correlate well with the performance of the descriptor employed by the recognition system. In addition to this, we evaluate several aspects of the matching task, including the efficiency of the different features, and the potential in using dimension reduction. To arrive at better generalization properties, we introduce a method for fusing several feature matches with a limited processing overhead. Our fused feature matches provide a significant increase in matching accuracy, which is consistent over all tested datasets. Finally, we benchmark all features in a 3D object recognition setting, providing further evidence of the advantage of fused features, both in terms of accuracy and efficiency.

## Background

This work concerns the problem of selecting an optimal local feature for certain estimation tasks. The seminal works of Mikolajczyk and Schmid (2005) and Mikolajczyk et al. (2005) have provided the basis for countless subsequent evaluations of interest point detectors and descriptors in images. In the 3D domain, local descriptors are an equally valuable mechanism for various estimation tasks, including object instance recognition and pose estimation. A very recent work (Guo et al. 2015) picks up the thread and provides a thorough performance evaluation of several 3D shape descriptors.

**Fig. 1:**
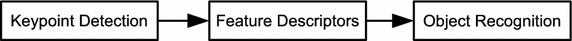
General structure of the processing pipeline of 3D object recognition systems

In this work we present an experimental design for evaluating various performance parameters relevant for the matching task. We base our evaluations on four datasets, relevant for both object recognition and wide baseline matching in a 3D setting. A large number of local shape features have been evaluated previously, however, most of these evaluations have primarily been focused on higher-level tasks such as pose estimation, model registration, and recognition (Aldoma et al. 2012; Bariya et al. 2012; Buch et al. 2013; Chen and Bhanu 2007; Chua and Jarvis 1997; Frome et al. 2004; Johnson and Hebert 1999; Jørgensen et al. 2015; Mian et al. 2006; Novatnack and Nishino 2008; Rusu et al. 2009; Stein and Medioni 1992; Zhong 2009). A recent work on 3D keypoint detector evaluation also exists (Tombari et al. 2013), providing a means to objectively determine the best algorithm for finding good feature points. Additionally, a number of recent works (Guo et al. 2013; Salti et al. 2014; Zaharescu et al. 2012) explicitly evaluate the performance of the introduced local descriptors. Although the latter two works base their evaluations on the same dataset, the dataset is modified in different ways, and the results show small variations. In Fig. 1 we boil the usual object recognition pipeline down to three individual steps. All three components have been well-studied in terms of performance evaluations; it is however not investigated what makes a feature good for a certain type of data. Indeed, as will show in this work, existing features do not generalize well across different datasets. The first part of this paper thus presents a unbiased comparison of different feature descriptors for different datasets, while the second part provides several new insights to the behavior of the different descriptors.

**Fig. 2:**
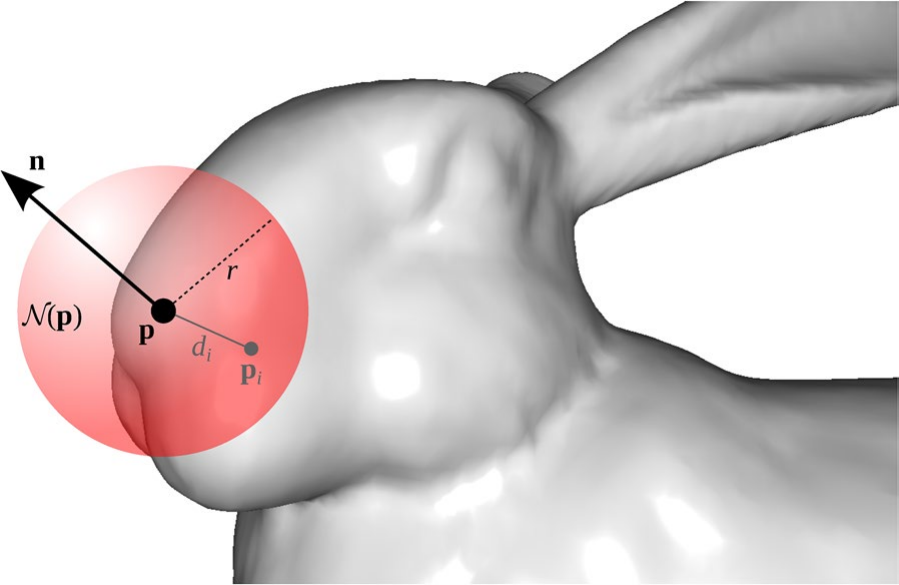
Geometric entities in a *spherical* support of radius $r$ around an oriented point $(\mathbf {p},\mathbf {n})$. Although many surface points exist in this neighborhood, we show only one, $\mathbf {p} _i$, at a distance $d _i$ from the *center*

For testing, we use four different datasets. Our work includes two popular object recognition datasets in our descriptor evaluations. That is, we do not only evaluate the recognition performance on these datasets, but also directly evaluate how the different descriptors perform on these datasets without the use of a recognition algorithm. Our tests result in a number of counter-intuitive findings—which we will elaborate on in "Matching accuracy" section—providing evidence that some of the descriptors currently regarded as the most accurate are showing a significant performance drop during this matching task, well below that of the classical less sophisticated descriptors. In addition to this, we show that using subspace representations, the performance of the best performing descriptors are virtually unaffected, even with a compression ratio above two. For boosting the matching performance with a limited processing overhead, we introduce a feature fusion algorithm, allowing for higher matching accuracy using existing features. Finally, we present a systematic benchmark for 3D object recognition scenarios using a baseline algorithm.

The contributions of this work are the following:

- A systematic and unbiased evaluation of local 3D shape descriptors.
- Evaluation results for several 3D datasets, including a synthetic dataset, two real-life datasets for object recognition, and a variable baseline scene matching dataset, which we have created based on a popular RGB-D dataset.
- Inclusion of our recently proposed local shape descriptor (Jørgensen et al. 2015), which is among the fastest features available, while providing a good trade-off between specificity and robustness.
- Additional evaluations of important feature characteristics: estimation and matching efficiency and the effect of using subspace representations for higher matching efficiency.
- A feature fusion algorithm for improving the matching accuracy based on a combination of different features.
- 3D object recognition benchmark results on real datasets, showing the relative performances of the tested features in a detection context, and the improvement gained by using our feature fusion algorithm.

**Fig. 3:**
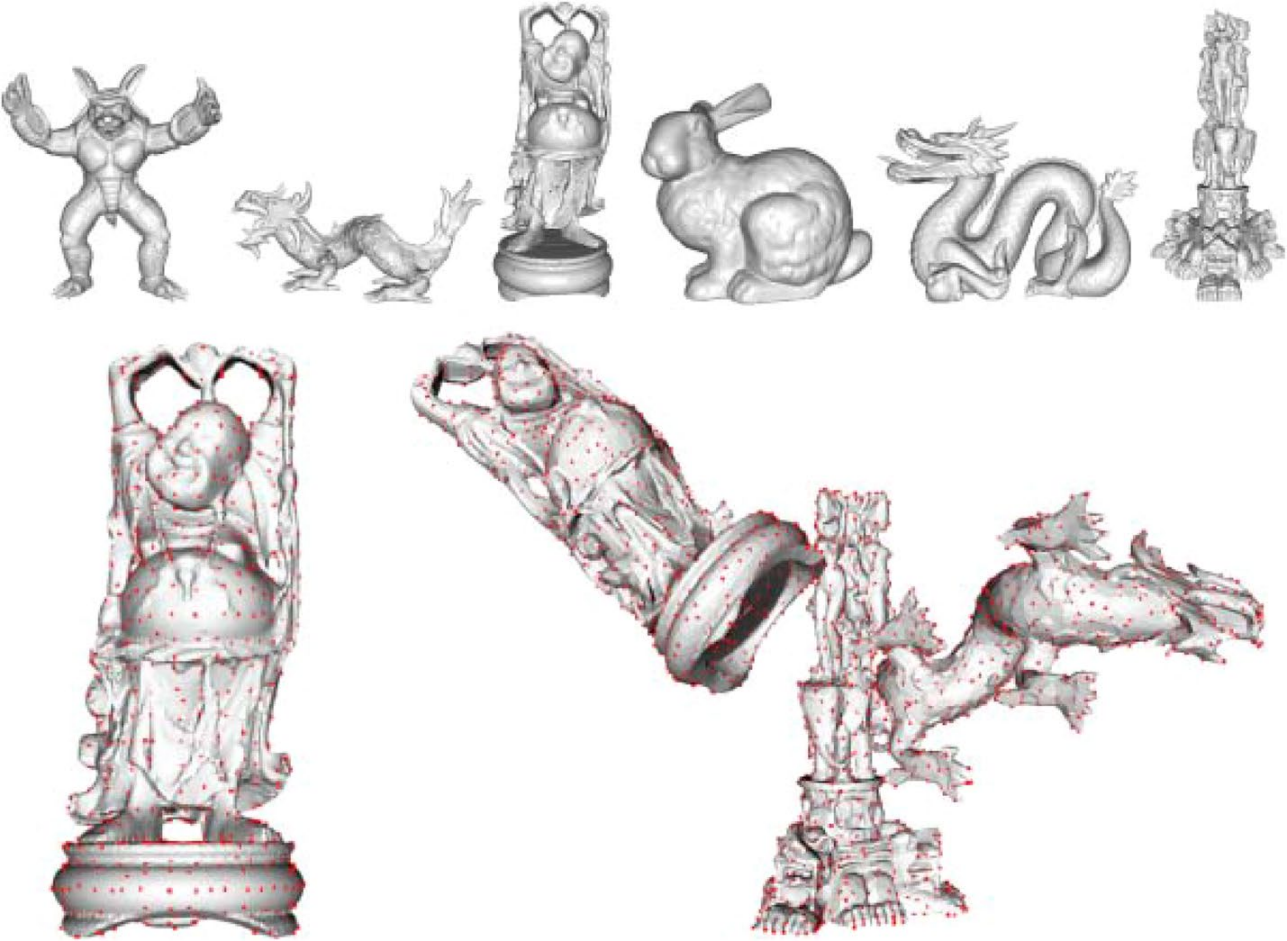
Bologna data.* Top* the six query models included in the Bologna datasets.* Bottom* uniform seed points for the *Buddha* model (*left*) and for the first scene in the Bologna 2 dataset (*right*)

**Fig. 4:**
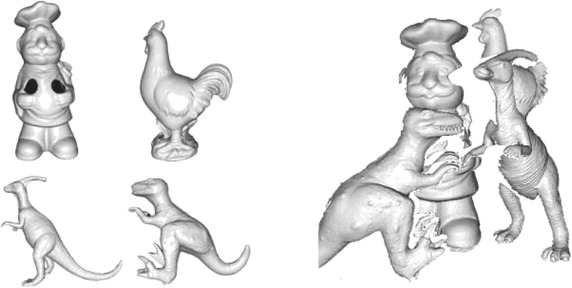
UWA data.* Left* the four query models included in the UWA dataset.* Right* the first laser scanner scene

This paper is structured as follows. In the following section (Related work), we outline previous works relevant for 3D feature evaluation. In "Feature descriptors" section all the evaluated descriptors are presented. The experimental protocol for all our experiments is given in "Experimental setup and datasets" section, along with an introduction to the datasets. "Matching accuracy" section presents the main evaluations of matching accuracy for all datasets. The following sections (Estimation and matching efficiency and The influence of the feature dimension) present evaluations of different feature aspects: efficiency during estimation and matching and the use of dimension reduction. In "Feature fusion" section we utilize our results to arrive at a feature fusion algorithm for improving matching accuracy. In 3D object recognition benchmarks we bring our contributions together in a systematic evaluation of the different features for 3D object recognition. Finally, we draw conclusions and outline directions for future work in Conclusion.

**Fig. 5:**
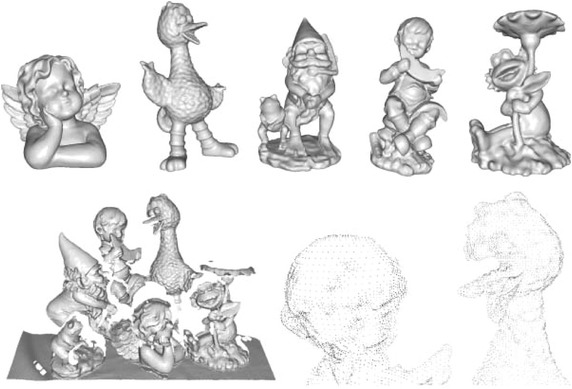
Queen’s data.* Top* the five query models of the Queen’s dataset.* Bottom* an example scene (*left*) of this dataset (scene 41) and a zoom (*right*) of the* upper region*, showing the variation of the local point density which occurs in this dataset

**Fig. 6:**
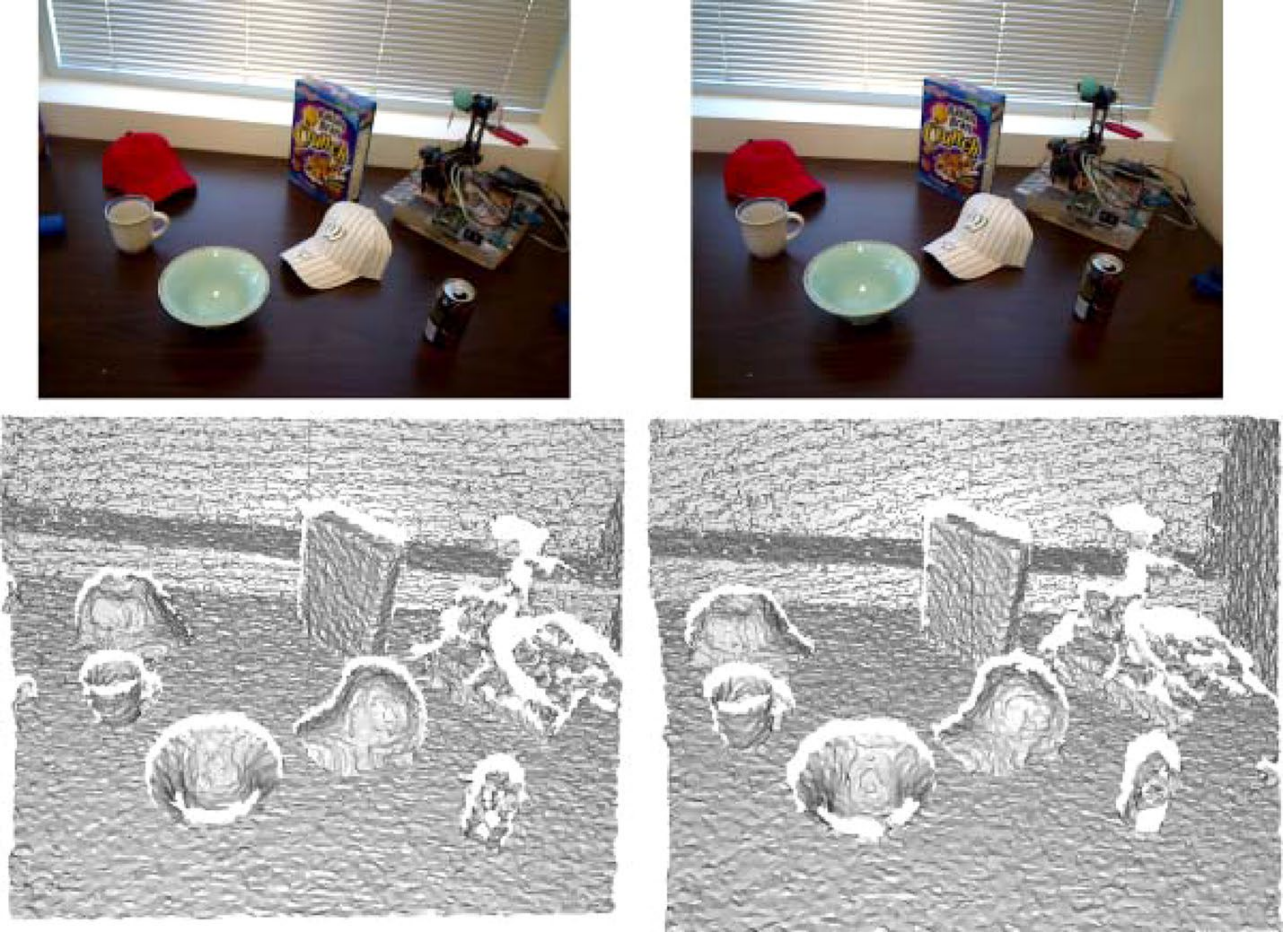
RGB-D Scenes data. A query/target scene pair from the *Table* sequence of the RGB-D Scenes dataset (frame 31 to the *left* and frame 36 to the *right*).* Top* RGB images.* Bottom* reconstructed meshes. Only depth information is used in our test scenes

## Related work

The most prominent works on feature benchmarking are most likely those of Mikolajczyk and Schmid (2005) and Mikolajczyk et al. (2005), providing comprehensive studies of both keypoint detectors and feature descriptors in images. Similarly, a newer study of Aanæs et al. (2012) extended the evaluation of keypoint detectors to different disturbances such as view point and illumination changes. Some of our contributions (Matching accuracy) adopt the same performance metrics as in these works, which however are restricted to wide baseline settings. We define suitable evaluation protocols for the object recognition datasets, where complete object models are matched against partial views.

The by far most frequent way of evaluating local shape descriptors is within different estimation pipelines, including model registration, pose estimation, and object recognition. An early work of Stein and Medioni (1992) extracts a combination of edge and surface features for object detection. The seminal work by Johnson and Hebert (1999) presents a recognition system based on the spin image descriptor. In Frome et al. (2004) the well-known 2D shape context descriptor (Belongie et al. 2002) was realized in a 3D version. Zhong (2009) used a more sophisticated spatial grid to increase the descriptive power of the descriptor, while Tombari et al. (2010) showed that by defining a unique and repeatable local reference frame, the descriptor achieved a significant performance boost. The 2D SURF descriptor (Bay et al. 2008) was also extended to 3D (Knopp et al. 2010) and applied for the task of shape classification.

**Fig. 7:**
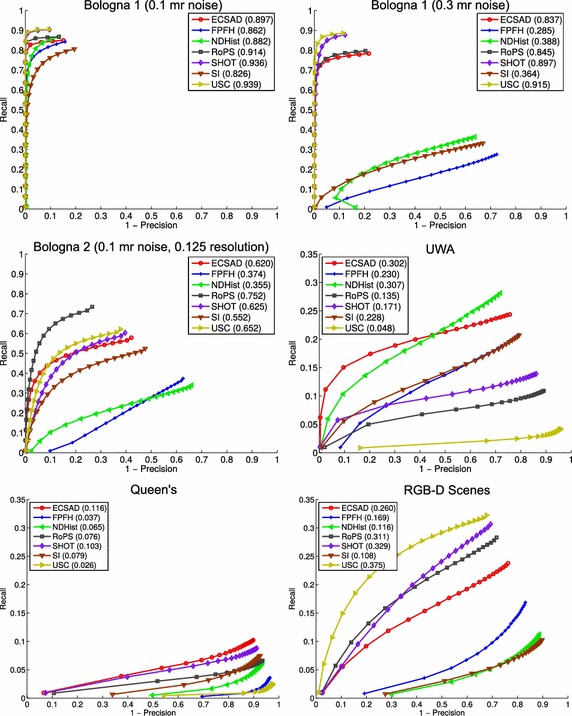
Matching accuracy results. The numbers in* parentheses* are the maximum $F_1$ scores computed over the curves

**Fig. 8:**
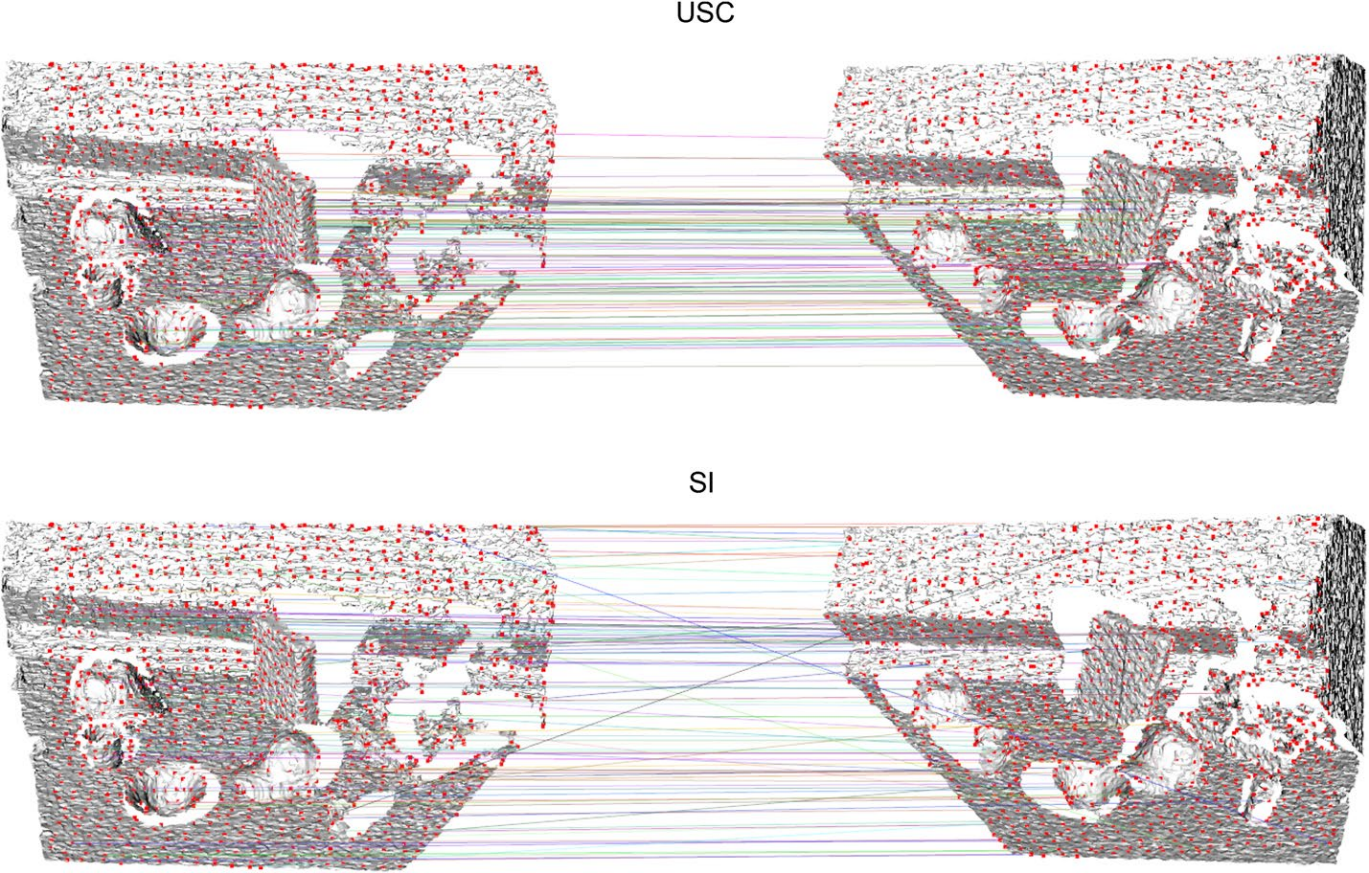
Correspondence example. The top 100 ranked correspondences of the best (*top*) and the worst (*bottom*) performing features of the RGB-D Scenes dataset, exemplified using the same scene as shown in Fig. 6. These figures also show the total set of seed points in *red*

Mian et al. (2006) introduced a full 3D modeling and recognition system based on local descriptors called tensors, with the addition of a now widely used object recognition dataset (the UWA dataset), which we will also use in this work. Later (Mian et al. 2010), the tensor representation was applied for scale-dependent recognition using a novel keypoint detector. Novatnack and Nishino (2007) defined scale-dependent operators for the detection of edges and corners in range images. Later, such features were applied for scale-dependent registration (Novatnack and Nishino 2008) and object recognition (Bariya et al. 2012). These features all require a range image as input, and are thus not directly applicable to 2D manifolds. Taati and Greenspan (2011) presented a number of variable-sized descriptors for higher flexibility, along with a new recognition dataset constructed in a manner similar to Mian et al. (2006). This dataset, the Queen’s dataset, is also considered in our experiments.

**Fig. 9:**
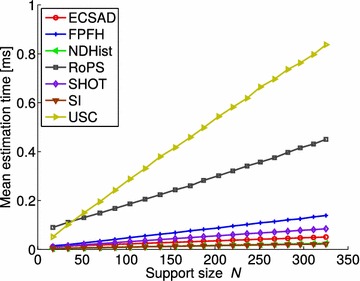
Descriptor estimation efficiency for different support sizes. Resolution vs. per-vertex feature estimation time. This *plot* is generated by keeping the support radius fixed while decimating the underlying surface, resulting in fewer neighbors

**Fig. 10:**
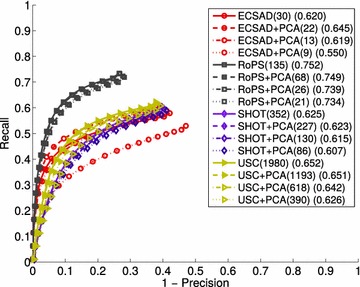
Effect of dimension reduction. Any feature vector can be compressed using PCA with minimal loss of accuracy, and in one case (ECSAD) even leading to increased accuracy. For each of the PCA-reduced features, the number of components is shown in the* first parenthesis*

In a notable work, Salti et al. (2014) presented a highly descriptive point cloud feature descriptor. This descriptor, and others, were evaluated on synthetic datasets, the Bologna datasets, which we will also include in our experiments. The same descriptor was used as a plug-in feature for sophisticated recognition pipelines in Aldoma et al. (2012) and Rodolà et al. (2013). Recently two descriptors operating on triangular meshes (Guo et al. 2013; Zaharescu et al. 2012) were presented. The former provided a general framework for describing not only shape information, but also various scalar fields on the manifolds, e.g. color. The latter exploited the mesh connectivity to increase the repeatability of local reference frames and presented a novel descriptor based on successive 2D projections of the point cloud in a local neighborhood. A recent survey (Guo et al. 2014) provides an extensive overview of current available methods for keypoint detection, feature description, and object recognition in 3D.

Out of all these works, we found several very different feature evaluations. Zaharescu et al. (2012) used, among others, the SHREC 2010 datasets (Bronstein et al. 2010), as well as a newly introduced benchmark. Similarly to the 2010 contest, the SHREC 2011 contest (Lian et al. 2011) also deals with shape retrieval of deformable objects. For these purposes, another class of features are tested, all with the aim of providing global descriptions of objects undergoing geometric deformations. These descriptors include Heat Kernel Signatures (Bronstein et al. 2011; Sun et al. 2009), Geodesic Distance Matrices (Smeets et al. 2009), meshSIFT Smeets et al. (2013), Features on Geodesics (Kawamura et al. 2012) and finally Shape-DNA (Reuter et al. 2006). We have chosen not to include such features and datasets, as they focus on non-rigid matching and matching of shapes with scalar fields, whereas we aim at evaluating 3D features for rigid shape matching, suitable for e.g. 3D object recognition and pose estimation. In Salti et al. (2014) a systematic descriptor matching evaluation is performed, primarily on the synthetic Bologna datasets. We use the same data here, but with a minor modification to the feature point selection phase, so as to remove the bias in choosing random points, which is done in the original work. Guo et al. (2013) applied the same synthetic dataset, but in different variations of noise and decimation. We revert to using the originally specified noise and decimation levels in our tests. For the specific task of evaluating 3D keypoint detectors, Tombari et al. (2013) applied, among others, the datasets from Salti et al. (2014), providing an extensive evaluation of 3D detectors and their performance on different data sources. Although the problem of 3D keypoint detection is very related to our work, we focus in our work solely on the feature matching stage, as we wish to quantify this aspect independently of any initial keypoint detection stage. We thus refer to the above very comprehensive works for more information on how to choose the best keypoint detector for different scenarios. The most recent and comprehensive work on 3D feature performance evaluations is Guo et al. (2015), which applies the same principles to many more descriptors and datasets. In a sense, we aim at collecting equivalent results for descriptors, but independently of any prior keypoint detection stage. Additionally, our experiments explicitly evaluate several different aspects of 3D descriptors, including descriptiveness, robustness, speed, and the complementariness of the shape information they capture.

**Fig. 11:**
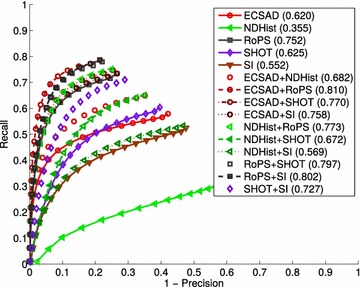
Effect of feature fusion. By the use of features in combination, a significant increase in matching accuracy can be achieved. Relative to the best performing single feature (RoPS), the binary combination ECSAD+RoPS achieves a 8 % increase in accuracy

**Fig. 12:**
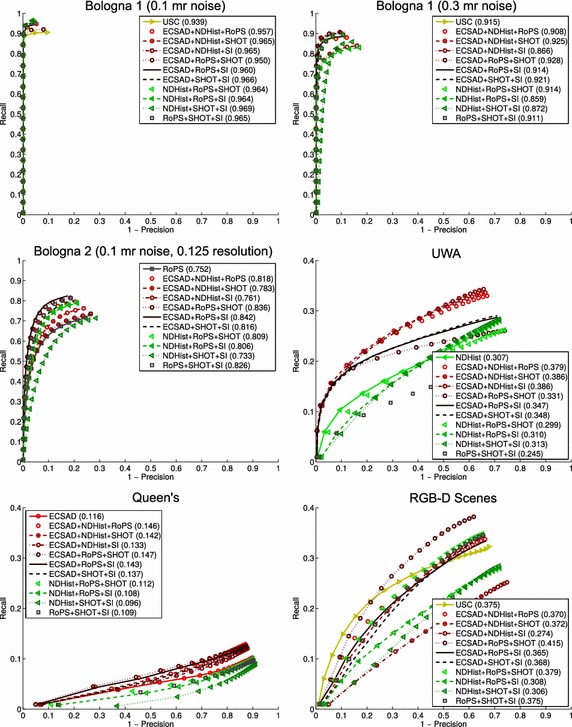
Matching accuracy results for all datasets using ternary feature fusion. Referring to the results in Fig. 7, we show the relative improvement of the best performing ternary feature over the best performing single feature for each dataset

## Feature descriptors

A vast number of local feature descriptors for 3D shapes have been presented over the last three decades, including Buch et al. (2013), Chen and Bhanu (2007), Chua and Jarvis (1997), Darom and Keller (2012), Frome et al. (2004), Guo et al. (2013), Johnson and Hebert (1999), Jørgensen et al. (2015), Mian et al. (2006), Novatnack and Nishino (2008), Rusu et al. (2009), Salti et al. (2014), Stein and Medioni (1992), Tombari et al. (2010), Zaharescu et al. (2012), Zhong (2009). The terms *feature* and *descriptor* can have different meanings in different computer vision fields. A common interpretation is that a feature refers to a point entity occurring at a distinguishable region in an image, e.g. an edge, a corner, or a blob. In the image domain features are therefore often the result of a detection stage such as the Canny edge detector (Canny 1986) or the Harris corner extractor (Harris and Stephens 1988). For matching tasks it is necessary to describe the features using an appropriate descriptor. For robustness towards occlusions and clutter, such a description is performed on a local scale using a neighborhood of pixels around the feature point. The well-known SIFT (Lowe 2004) and SURF (Bay et al. 2008) algorithms both come with dedicated interest point detectors (difference of Gaussian and fast Hessian) and descriptors (weighted gradient and Haar wavelet histograms). In 3D the principle remains the same, but the detection step is often omitted and replaced simply by a uniform or random sampling on the surface of the shape (Aldoma et al. 2012; Salti et al. 2014). The descriptor is computed in a spatial neighborhood around the feature point. In the rest of this paper we use *feature point* to refer to the point which is being described, and *feature vector* or *descriptor* for the often histogram-based description of this point based on the local spatial neighborhood.

**Fig. 13:**
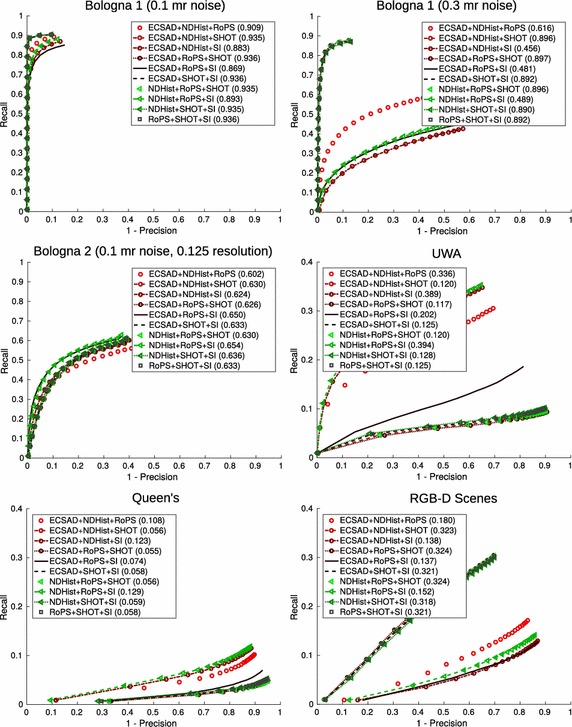
Matching accuracy results for all datasets using feature fusion at feature-level, as proposed by Lei et al. (2013)

**Fig. 14:**
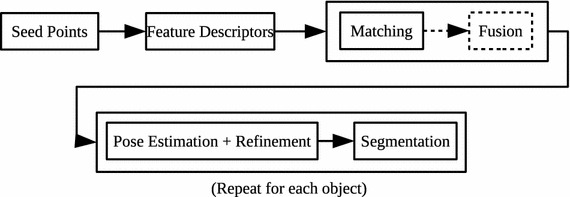
Object recognition diagram for an input scene

In the sequel, we introduce the features used for testing in this work. For notation purposes, we use the symbols tabulated in Table 1. Some of these are also visualized in Fig. 2.

**Table 1:** List of geometric parameters for feature estimation

Symbol	Description
$\mathbf {p}$	3D point
$\mathbf {n}$	3D normal vector
$d$	Euclidean or $L_2$ distance
$r$	3D support radius for computing descriptors
$\mathcal {N} (\mathbf {p})$	Set of neighbors in the support of $\mathbf {p}$
$N =\left| \mathcal {N} (\mathbf {p})\right|$	Number of neighbors or support size
$\mathbf {f}$	Feature vector or descriptor

### Spin Image (SI)

The SI descriptor represents an early example of successfully applying local descriptors for 3D object description and recognition (Johnson and Hebert 1999). Each neighbor within the support of an oriented point are described by the cylindrical coordinates $\left( \alpha ,\beta \right)$. The $\alpha$ coordinate is the radial distance, measured as the perpendicular distance from the neighbor point to the line through $\mathbf {n}$. The $\beta$ coordinate is the signed point to plane distance from the neighbor point to the tangent plane defined by $\mathbf {p}$ and $\mathbf {n}$. All $\left( \alpha ,\beta \right)$ pairs are binned in a 2D histogram with bilinear interpolation for increased stability towards noise. We have tested different binnings in $\left( \alpha ,\beta \right)$-space and found good performance for 9 radial and 17 elevation bins, giving a 153-dimensional SI descriptor.

**Fig. 15:**
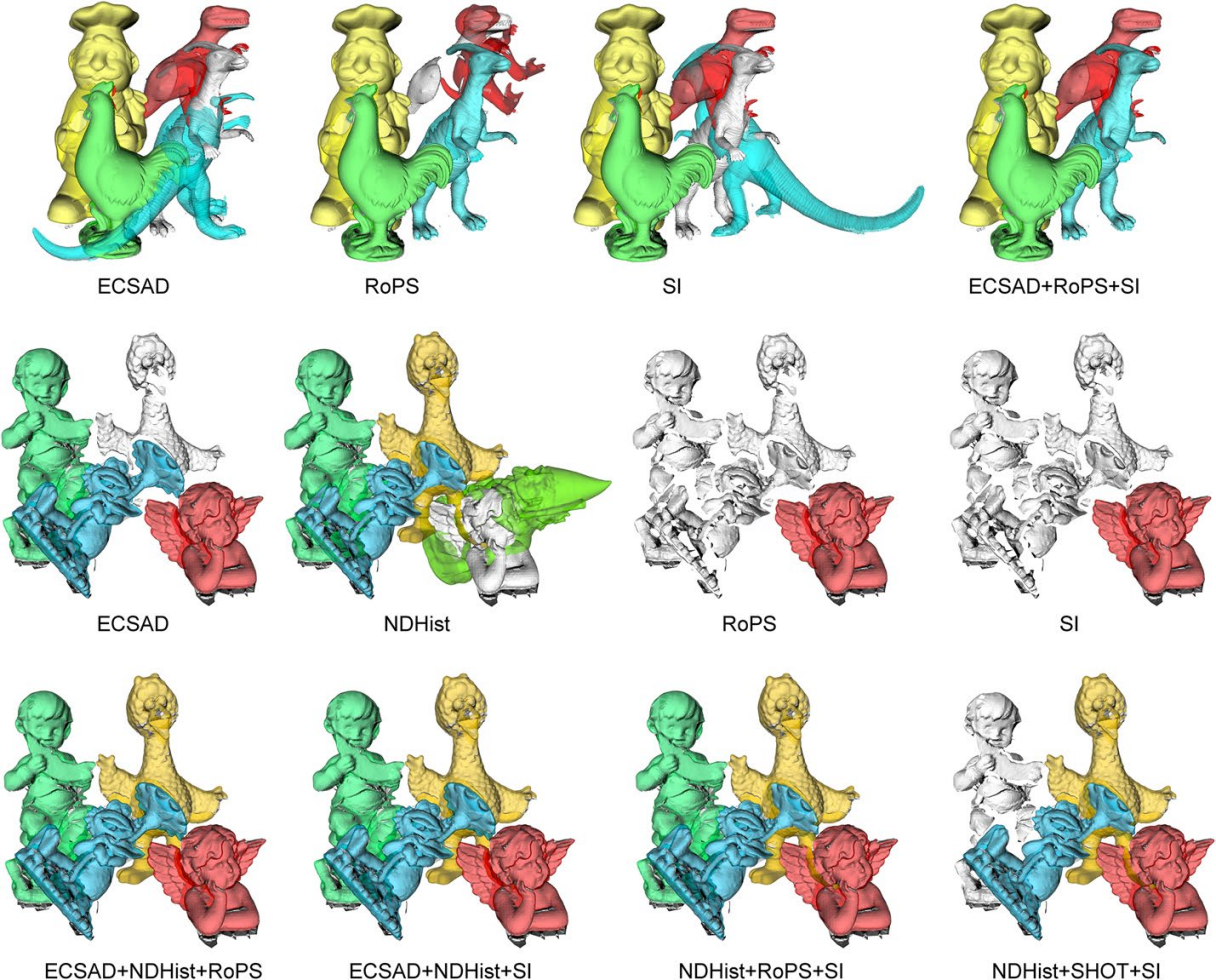
Qualitative recognition results using single vs. ternary features. Detected objects are overlaid with* different colors*.* Top row* UWA scene 3, where the single features ECSAD, RoPS, and SI fail individually, but their ternary combination succeeds.* Middle row* Queen’s scene 22, where most of the single features show suboptimal results [false negatives in (e)–(h) and a false positive in (f)].* Bottom row* all ten ternary combinations except NDHist+SHOT+SI (only four shown) detect all objects

### Fast Point Feature Histogram (FPFH)

The FPFH descriptor (Rusu et al. 2009) requires two sweeps through all feature points on the surface. In the first sweep, each oriented point $(\mathbf {p},\mathbf {n})$ is paired with each of its oriented neighbors $(\mathbf {p} _i,\mathbf {n} _i)$, and a local Darboux frame is used for computing three angular measurements, which are used for computing three *simple* histograms. In the second sweep the distance-weighted sum of all histograms in the support is computed to build the final 33-dimensional FPFH descriptor.

### Signature Histogram of Orientations (SHOT)

The SHOT feature (Salti et al. 2014) is arguably the first feature to make use of a unique and repeatable local reference frame (LRF), which can be seen as a 3D generalization of the dominant orientation estimation employed by e.g. SIFT. The LRF is—similar to SIFT—used for partitioning the local support, and for each spatial region a histogram of relative orientation (normal) cosines. The descriptor uses 32 spatial regions, each with an 11-dimensional histogram, leading to a relatively high-dimensional feature vector of length 352.

### Unique Shape Context (USC)

USC (Tombari et al. 2010) is a development of the 3D Shape Context (Frome et al. 2004), which is a 3D adaption of the well-known Shape Context edge descriptor in 2D (Belongie et al. 2002). The improvement in USC is achieved by replacing the non-unique LRF by the exact same LRF computation technique as in their previously proposed SHOT feature. For USC each spatial region contains a single density-normalized scalar, and the descriptor dimension is thus directly given by the number of spatial bins, which is quite high: 15 radial, 12 azimuth and 11 elevation regions, giving 1980.

### Rotational Projection Statistics (RoPS)

RoPS (Guo et al. 2013) is a recent descriptor, which applies some of the same principles as SHOT for LRF computation, but uses a fundamentally different approach for description. It is the only descriptor we have tested that also requires a triangle mesh, and not just a point cloud. The mesh connectivity is exploited for increasing the repeatability of the LRF. 15-dimensional histograms are computed by a series of projections of rotated versions of the local point cloud onto the local planes. The information in each histogram is condensed to five statistics by computing the first four Hu moments and the Shannon entropy. There are a total of nine rotations (three per axis), giving a final feature vector with 135 components.

### Equivalent Circumference Surface Angle Descriptor (ECSAD)

The most recent descriptor is the ECSAD, originally developed for the purpose of detecting edges at orientation discontinuities in point clouds (Jørgensen et al. 2015). ECSAD splits the local region along the radial and azimuth axes, but not elevation, in order to reduce the number of empty regions. For each neighbor point in the support $\mathbf {p} _i$ the relative angle between $\mathbf {n}$ and $\mathbf {p} _i-\mathbf {p}$ is computed. All angle measurements that fall into the same spatial bin are averaged, and an interpolation scheme is employed to fill in missing values in empty spatial bins. ECSAD has a relatively low dimension of 30.

### Normal vs. Distance Histogram (NDHist)

The NDHist descriptor is a simple and fast baseline descriptor which we have developed for our experiments, inspired by the works of Buch et al. (2013) and Mustafa et al. (2015). For a given feature point, we compute the relative Euclidean distance and the dot product of the normals for all neighbors in the support. For an oriented feature point $(\mathbf {p},\mathbf {n})$, we thus get $N$ pairs of $\left( d _i,\mathbf {n} \cdot \mathbf {n} _i\right)$ measurements. A relative 2D histogram of distance vs. normal dot product is then generated from these samples. We tuned the bin numbers along the two axes and found 8 distance bins vs. 16 normal bins to achieve good performance, giving a feature vector of length 128.

### Commonalities and differences

All the descriptors mentioned above aim at capturing complete information of the local surface variation, but in very different ways. Except for FPFH, they are all based on some spatial decomposition, which is used for binning the one- or two-dimensional histograms. For FPFH, the binning in the descriptor is purely done over the domain of the angle measurements. The NDHist is special in the sense that it uses spatial bins (relative distances) vs. bins over the domain (relative angle measurements). For SI and ECSAD the decomposition is performed using different radial and/or elevation binnings, and they are both designed to be rotationally invariant to the angle around the surface normal—in the SI case the descriptor is $\infty$-fold rotationally symmetric, in the ECSAD case the rotational symmetry is twofold. The binning of these descriptors is directly given by the spatial bins. The same holds for USC, which however has no rotational integration due to the unique LRF. Finally, SHOT and RoPS aim at combining the best of both worlds by using on the one hand a coarser spatial decomposition, and on the other hand a small histogram within each spatial region.

When considering feature matching performances, there is a trade-off between *specificity*^1^ and *robustness*. We use specificity to refer to distinctiveness, i.e. the ability of a feature descriptor to distinguish between different regions with almost similar appearance. In the presence of perturbations caused by e.g. noise and varying density, local regions change appearance. If we consider the set of possible descriptors of a feature as a high-dimensional manifold,^2^ such perturbations cause a displacement of the feature vector on the manifold. However, the discrete binning that is performed while building a histogram-based descriptor causes a discretization of this manifold, which prevents movement along the manifold under small perturbations. Thus, if a feature is robust, the discretization of this manifold is coarser, allowing for more perturbations. In other words, the feature is able to maintain invariance to a higher degree of perturbations. All LRF-based descriptors (USC, SHOT and RoPS) are expected to show a higher degree of specificity, but at the expense of a decreased robustness in the presence of noise. Additionally, as we will show in "Matching accuracy" section, these descriptors significantly degrade under occlusions, which will occur when a full model is matched with a scene view. For a comprehensive overview of different feature characteristics, we also refer to the analysis in Salti et al. (2014).

All features except ECSAD and NDHist are available in the Point Cloud Library.^3^ The code for computing ECSAD and NDHist descriptors are available in the CoViS project.^4^

## Experimental setup and datasets

This section describes the experimental protocol which we have defined for performing all experiments in the following sections. The basic task is to match local 3D structures between two models. The first model we term the *query* model, and the second model the *target*. For wide baseline tests, where two scene views are matched, both query and target come from a view of the same scene. When testing on object recognition datasets, the query model represents a full object, and the target is a scene containing zero or more instances of the object. For the synthetic scenes we consider, the target scenes contain the full object models, corrupted by noise and decimation, but for real scenes only partial data of the object is visible due to occlusions. For all target scenes in all of our datasets, we have ground truth pose information, i.e. the *SE*(3) transformation required to bring one or more query models into alignment with the target data.

### Methodology

For testing a single query-target pair of models, we loosely follow the experimental protocol of Salti et al. (2014), but with a few modifications, and extensions to allow for testing the features in scenes with occlusions. The steps in our evaluation protocol are described in the following.

#### Feature seed points

In Salti et al. (2014) the query models in the dataset are randomly sampled to get 1000 feature points per model at random locations on the surface, and these surface points are described using the descriptors available. These points are also known as *seed points*. For each target model, the seed points of each query model are placed in the scene using the ground truth pose, and the target seed points are now described. A scene with e.g. three complete objects will thus contain 3000 seed points. We propose a slightly different approach for sampling seed points. Instead of random sampling, we use a voxel grid (Rusu and Cousins 2011) to get a uniform sampling of the query surface by taking the surface point nearest to the center of each non-empty voxel. The voxel size is tuned so that it produces approximately 1000 query points on average. This ensures that all results are reproducible.

In our experiments, we will encounter scenarios where a query model is matched against an incomplete instance of itself in the target model. In these cases, it is not possible to find any matches for the occluded parts in the target. Including these occluded parts would introduce a high amount of negative examples, which is not desirable, as this would shadow the actual precision of a feature during matching. Similar to Mikolajczyk and Schmid (2005), we thus remove non-overlapping regions and discard missing seed points in these situations. This is implemented simply by checking if a transformed query seed point has a neighbor in the target up to the resolution of the target.

#### Feature description

Upon selection of seed points, we pass the points to be described to the different descriptor estimation routines. Generally, the matching accuracy of a descriptor is expected to increase with increasing support radius, except in cases where occlusions and clutter are present. For fair comparisons we use the individually tuned support radii for all descriptors shown in Table 2, specified as a multiple of the average mesh resolution (mr) of all the query models. The mesh resolution is computed as the mean edge length of the mesh.

**Table 2:** Tuned feature descriptor radii

Feature	Bologna	UWA	Queen’s	RGB-D scenes
ECSAD	30	10	10	25
FPFH	20	7.5	7.5	17.5
NDHist	30	20	22.5	25
RoPS	30	12.5	12.5	20
SHOT	30	17.5	20	25
SI	30	10	12.5	22.5
USC	30	12.5	12.5	25

All numbers are given as multiples of the average mesh resolution

During descriptor computation, the underlying mesh is utilized, in some cases in a decimated version (see Datasets). The normal orientation of each surface point is computed by the area weighted mean of the incident mesh triangles (Thürrner and Wüthrich 1998). For fast and exact localization of all points within the support radius $r$, *k*-d trees are used (Muja and Lowe 2014).

#### Feature matching

At this stage, we have descriptors computed for a target model and corresponding descriptors for one or more query models appearing in the target scene. We now use a brute-force linear search for the nearest matching features of each query feature in the target model. We have tested the use of many different distance functions during this stage, including $L_1$, $L_2$, and $L_\infty$, but also distribution distances such as $\chi ^2$, the Hellinger distance, and the Earth Mover’s Distance (Rubner et al. 1998). The by far best results were achieved with the $L_2$ distance, which is why we restrict ourselves to presenting results for this metric. Additionally, multiple studies have shown the advantage during matching of taking the ratio of the nearest matching feature distance to that of the second-nearest match (Lowe 2004; Mikolajczyk and Schmid 2005). Our initial analyses confirmed the superior performance of this matching strategy, and we thus employ this strategy in all results to follow. The overhead of finding two nearest neighbors instead of one is negligible and leads to an increase of less than 1 % in search time.

We note that for practical applications, approximate search techniques for finding nearest features in high dimensional spaces can provide magnitudes of speedup with limited loss of precision [e.g. Muja and Lowe (2014); Nene and Nayar (1997)]. For our benchmarks we find exact neighbors by a brute-force search, whereas in our recognition experiments in 3D object recognition benchmarks we use approximate searches for speedup.

#### Evaluation of matches

Once the complete set of matches have been established, they are ranked according to the $L_2$ distance ratio. One dataset contains many target models, each with one or more query models. All matches over all models are collected in a single sorted array. Then we vary the upper matching threshold by traversing the sorted array and count the number of correct matches in the current subset. As per previous descriptor evaluations (Mikolajczyk and Schmid 2005), we present all results as 1-precision vs. recall (PR) curves. Precision refers in this context to the number of correct matches to the total number of matches at a given distance threshold. Recall refers to the number of correct matches at a given distance threshold to the total number of possible correct matches (i.e. the number of feature seed points in the target). To provide a single quantitative and conservative measure of the overall *accuracy* of a feature, we associate to each PR curve the maximum $F_1$ score, which is computed as the maximal harmonic mean over all (*P*, *R*) observations along the curve:

$$\begin{aligned} \max F_1 = \max _{(P, \, R)} \left( 2\cdot \frac{P \cdot R}{P + R}\right) \end{aligned}$$

Since the distance ratio ranking maintains the nearest neighbor match of each feature, the final precision and recall at the end of the curve is the same as it would be if using only the nearest matching feature distance for ranking. It is only the precision along the PR curve and thereby the $\max F_1$ score that is increased, as the distance ratio provides a better internal ranking than the nearest neighbor distance. In "Feature fusion" section we will present a matching strategy for increasing both precision and recall by the use of feature fusion.

### Datasets

For our purposes, we have considered four different datasets from different data sources. These are all described below.

#### Bologna 1 and 2

The Bologna dataset (Salti et al. 2014) is a collection of six full object models and 45 synthetic scenes, generated by applying a random rigid transformation to a random subset of the objects. These object models are taken from The Stanford 3D Scanning Repository.^5^ The synthetic scenes are now altered by isotropic Gaussian point noise and by mesh decimation. The *Bologna 1* dataset refers to two noisy versions of the 45 scenes (0.1 and 0.3 mr), and *Bologna 2* refers to a noisy and decimated version of the 45 scenes (0.1 mr noise followed by a decimation factor of 0.125). The Bologna 2 dataset thus allows for testing the robustness of a descriptor towards different point densities, as it only contains approximately 12.5 % of the number of vertices in the query models. A visualization of the six models and the seed point selection mechanism is shown in Fig. 3.

#### UWA

The UWA object recognition dataset (Mian et al. 2006, 2010) is composed of four full object models, generated by a multi-view registration algorithm, and 50 real scenes captured with a laser scanner containing incomplete instances of the objects (see Fig. 4). This dataset is heavily used in the literature as the benchmark for 3D object recognition systems, as it allows for testing such systems in a realistic environment. Contrary to the Bologna datasets, where the ground truth pose was given by design, the ground truth poses of the objects have been obtained manually for this dataset. On average, the objects and scenes of the UWA dataset contain more than 100,000 mesh vertices, as they come directly from the raw sensor data. This makes the models quite impractical for description, both for efficiency reasons and due to the high noise levels. Similar to previous works (Mian et al. 2006), all objects and scenes are thus decimated down to a factor of 0.125 (Garland and Heckbert 1997) to produce high-quality meshes using MeshLab.^6^ We again tune the voxel size for the seed selection to produce approximately 1000 seed points per object.

Contrary to the Bologna scenes, the scenes of UWA are not in complete correspondence with the query models due to the high levels of occlusion. For descriptor matching, it is therefore natural to reverse the direction, i.e. to find the nearest neighbor within the query model set for each of the target features. This is the approach taken in many successful object recognition algorithms tested on this dataset (Aldoma et al. 2012; Drost et al. 2010; Guo et al. 2013; Mian et al. 2006; Papazov and Burschka 2011; Rodolà et al. 2013). By this approach, it is still possible for a feature to achieve full recall, since each scene feature will always have a ground truth match in the object library. Using full object to partial scene matching would introduce a high amount of negative examples, which would make the results incompatible with the other datasets, which do not contain true negatives.

#### Queen’s

The Queen’s dataset (Taati and Greenspan 2011) is constructed in completely the same manner as the UWA dataset and consists of five object models and 80 scene models captured with a laser scanner. Compared to the UWA dataset, the Queen’s scenes have a larger variation in the number of objects present (between one and five), and the scenes are of lower quality as the local point density varies. Like the UWA dataset, for this dataset we also employ a reverse matching strategy from scene features to the object library.

For this dataset the query objects and target scenes are all provided as point clouds, so we reconstruct a mesh from them to be able to compute RoPS descriptors. The scenes can easily be triangulated by exploiting the inherent 2D grid structure of the points. Each 3D point is simply normalized by its depth, and the depth component is discarded, resulting in a 2D grid, which is converted to a mesh using Delaunay triangulation. The object models are triangulated using the MeshLab implementation of the Marching Cubes algorithm (Guennebaud and Gross 2007) with a grid resolution of 200. This, however, increases the resolution the models, and we therefore apply mesh decimation (Garland and Heckbert 1997) to the triangulated models with the decimation factor tuned for each model to restore approximately the same number of vertices as in the input point cloud. To arrive at a reasonable resolution in the scenes, we decimate them with a factor of 0.75. A visualization of reconstructed meshes from this dataset is shown in Fig. 5.

#### RGB-D Scenes

The RGB-D Scenes come out of the larger RGB-D Dataset (Lai et al. 2011, 2012) and contains eight video sequences of indoor scenes captured with the Kinect. Each sequence contains thousands of frames, which have all been aligned by an RGB-D mapping algorithm. For each frame, an accurate camera pose relative to the first frame is thus provided. Based on these sequences, we have generated a dataset suitable for descriptor matching experiments. From each sequence, we first take every fifth frame and discard the intermediate frames. Now every second frame is considered a query model and the other frames target models, giving a large number of query/target pairs, each of them five frames from each other. In a sense, our dataset is equivalent to the wide baseline benchmark provided by Mikolajczyk and Schmid (2005), but with many more frame pairs—143 for our dataset. Additionally, our scene pairs have varying spatial separation and motion blur due to varying camera velocities, and varying difficulties, since they contain both planar structures (tables, walls) and distinctive regions (objects from the RGB-D dataset).

Similar to the Queen’s dataset, the scenes are converted to a mesh using a 2D Delaunay triangulation on the depth images, before reconstructing the whole mesh to 3D using the known focal length of the Kinect sensor. Finally, the scenes are decimated with a factor of 0.125 to reduce noise and to keep the number of surface points at a manageable level. An example frame pair is shown in Fig. 6, which shows one of the easier scenarios with a limited baseline and many objects. Similar to the Bologna dataset, for our RGB-D Scenes there is a good correspondence between the query and the target, due to the fact that they are obtained from the same sensor with limited movement between the frames. However, being captured with a low-cost Kinect sensor, this data contains a considerable amount of noise. This combined with the high amount of planar structures makes this dataset favor descriptors that have both high specificity and robustness.

#### Feature-specific parameters

During feature descriptor estimation, we must choose a support radius $r$ based on a trade-off between the desired level of descriptiveness and robustness towards occlusions. For different features the optimal radius may vary, and we have tuned this external parameter to squeeze out the best performance of all the descriptors. This radius is set as a multiple of the average resolution computed over all the query meshes, and we have tested all possible radii in the range $\lbrace 5,7.5,\ldots ,30\rbrace$ mr. For the Bologna scenes, we used the Bologna 2 dataset to tune this parameter, as it represents the most difficult version of the scenes. The final tuned radii are shown in Table 2. Expectedly, the FPFH requires a small radius, since its effective influence radius is $2r$. USC requires an extra parameter specifying the radius to use for estimating the local point density. This was also tuned, and we achieved the best results with this radius set to 2 mr for all datasets.

## Matching accuracy

We now present the feature matching results produced by our benchmark. These are presented in Fig. 7 using PR curves with the $\max F_1$ scores shown in parentheses right next to the feature names. The points along the curves are sampled in such a way that there is an equal amount of data points between two samples. This reveals the additional property that most of the curves contain a majority of samples with a low precision. The scores are shown with three significant digits, since some results differ by very small amounts. This is reasonable, because the curves, and thereby the scores, are computed based on thousands of data points. The same holds for all the results presented in the rest of this paper.

The results for the Bologna datasets in Fig. 7 are comparable with the results of the original work (Salti et al. 2014). We did, however, get better results for SI, most likely because we used another bin size. The rest of the discrepancies in our results are minor and most likely due to the differences in the seed point sampling. From these three datasets, we observe that the newer LRF-based features (ECSAD, RoPS, SHOT, and USC) show superior performance. At the lowest noise level for Bologna 1, the performance of NDHist comes near these descriptors, and for the Bologna 2 set the SI descriptor shows almost competitive performance with ECSAD, SHOT, and USC. For the remaining results in all three Bologna datasets, the simpler histogram features (FPFH, NDHist, and SI) show poor performances, which should be attributed to their low specificity. For the noisy Bologna 1 scenes, USC and SHOT provide superior results, while for the Bologna 2 set the best descriptor is RoPS. This difference in results may be explained either by the use of the mesh structure in RoPS, which makes it more robust to density variations, or by the simple fact that the RoPS descriptor has a coarser resolution, making it more robust in general. The latter hypothesis is, however, not supported by the results for the UWA and Queen’s dataset, which we will describe shortly. We believe even more systematic evaluations of the influence of these factors are required to determine exactly why the performances of RoPS vs. SHOT and USC switch places, but this is beyond the scope of this work. To reiterate, the results of the Bologna datasets are along the lines of recent developments and have been used as a solid argumentation for the use of recent features (Guo et al. 2013; Salti et al. 2014).

One of the main findings of this work becomes evident when we look at the results for the UWA object recognition dataset, which is frequently applied for benchmarking descriptor-based recognition systems. Here we see almost reverse performances compared to the Bologna results. The best descriptor is the simplistic NDHist, closely followed by ECSAD. These performances are followed by those of FPFH and SI. The best feature during the tests of the Bologna 1 datasets, USC, is the worst performer for this dataset. We believe that this result highlights a fundamental problem with the recently developed features, namely that they are designed primarily towards specificity, by which too much robustness is sacrificed. The only LRF-based descriptor which shows good performance is ECSAD, arguably because it is designed to be invariant to the sign of the x-axis of the LRF. This is supported by the general tendency that ECSAD performs above average on all datasets. The robustness problems with the LRF-based features are therefore caused either by instabilities in the sign disambiguation stage, or by their high resolution. The poor results of USC speak for the latter explanation, but we believe that the high levels of clutter and occlusions in the scene models also makes it impossible to define a repeatable LRF, when the query models consist of full models without these disturbances.

Looking further at the Queen’s results, we see an overall poor performance, as this dataset is particularly challenging. It is in our view hard to determine whether ECSAD and SHOT actually show good performances here, or whether the scale of the curves is so low that the relative performances are negligible. Opting for the first possibility, the robustness of SHOT towards varying sampling density may explain its relatively high performance. This would also explain the performance drop for NDHist, which is expected to be very sensitive to this factor.

Finally, the results of the RGB-D Scenes are more clear, and favor the LRF-based descriptors. This can be attributed to the fact that the scene pairs in this dataset are in good correspondence, and that they have an almost equal resolution. Under these circumstances, it seems that the LRF-based descriptors are very good at tolerating noise, primarily in depth, as it originates from a real sensor. A qualitative result for this dataset is shown in Fig. 8.

From all of these results, we draw the conclusion that *none of the features shows good generalization properties*. Although ECSAD is in general a high performer (especially for UWA and Queen’s), it is not on par with the best results for the Bologna 2 set and the RGB-D Scenes set. We believe that one explanation to this problem lies in the fact that the different features aim at capturing very different aspects of the local appearance of a shape. For a feature such as USC, the aim is clearly to capture even the smallest variations in depth values, giving high responsiveness to high frequency content. A feature such as SI, on the other hand, is designed to provide a smooth and complete image of the local support, giving a low frequency signal in the output descriptor. In the following section, we evaluate more aspects of the performances of these features, but we return to addressing this problem in "Feature fusion" section, where we will present one solution for arriving at good generalization properties.

## Estimation and matching efficiency

A crucial aspect of recognition pipelines based on 3D feature estimation and matching is the efficiency during these two processes. In many applications, the feature matching step should be finished quickly, before a more sophisticated recognition algorithm processes these matches to produce detections. In this section, we benchmark the different features, both in terms of the time spent for estimating the features, and in terms of the time spent on matching them. All benchmarks are, unless otherwise noted, performed in single-threaded processes on a desktop computer equipped with a Intel Xeon E3-1245 v2, 3.4 GHz processor.

### Estimation efficiency

For assessing the efficiency during feature estimation, we examine the complexity of this process when the number of neighbors in $\mathcal {N}$ increases linearly. A linear increase in $N$ can be achieved either by increasing the radius $r$ with $\sqrt{2}$ (the local surface patch within a small $r$ on a surface manifold is approximately 2D, so $N$ increases with the circle area $\pi r ^2$), or by linearly increasing the resolution of the surface under a fixed $r$. Both methods are completely valid, and we have made a choice on the latter option. We thus decimated the first scene in the Bologna 1 dataset to 20 resolution levels: $\lbrace 0.05, 0.1, \ldots , 1\rbrace$, giving a linear increase in the average value of $N$ from 17 to 325. Then features are computed at every data point in the decimated scenes with $r =0.01\,\hbox {m}$, corresponding to a factor 7.5 of the average object mr, which is significantly smaller than the optimally tuned values in Table 2 (the next section explains why we use a small radius here). The results, shown as the support size vs. the mean per-vertex estimation time, are plotted in Fig. 9. All timings in this figure include the *k*-d tree based radius search for neighbors, which is equal for all features. In the special case of the RoPS feature, a mesh is required. This is already available for the Bologna scenes, but for point cloud data, mesh triangulation would be required as a preprocessing step, potentially causing a significant computational overhead.

The by far fastest features are the histogram-based SI and NDHist, which require a simple sweep through all the neighbors within the support (projection followed by accumulation). The ECSAD feature spends twice the amount of time per vertex, possibly due to the high number of internal branches and arctan calls required for the angle computations. The SHOT feature shows an impressive estimation efficiency in spite of its complexity. This is followed by FPFH, for which we see the penalty of using two sweeps over the surface. The RoPS and USC features are significantly slower than all other features. For RoPS, the explanation lies in its complexity, both caused by the many area computations and the processing of the many local projections. For USC, each neighbor of a feature point contributes to a bin count by a density-normalized value, which requires for that neighbor a local search, leading to an expensive descriptor computation.

### Matching efficiency

Here we investigate the effect of the dimension of a feature descriptor on the efficiency during matching. We base our results on the same decimated Bologna 1 scenes as in the previous section, but now using only the feature seed points. Each decimated scene thus contains $\sim$2500 feature points, and the task is to match each of the object model features against all of these candidate matches per scene. In principle any of the datasets could be used for this experiment, as the dimension of all the features are fixed. Note also that the number of candidate matches influences the results, so we consider relative performances. As in all previous experiments, we carry out a linear search for the two nearest features. The results are reported in Table 3.

**Table 3:** Matching efficiency results for all features, with the dimension shown in parentheses

Feature (dim)	Matching time (ms)	Speedup
USC (1980)	2.766	1.00
SHOT (352)	0.491	5.64
SI (153)	0.226	12.3
RoPS (135)	0.202	13.7
NDHist (128)	0.194	14.3
FPFH (33)	0.061	45.4
ECSAD (30)	0.056	49.5

All numbers in the middle column give the mean per-vertex time [ms] for a linear search for the two nearest feature neighbors

If we consider the results at full resolution in Fig. 9 ($N = 325$) along with these results, we notice that for most features the per-vertex matching time is close to or higher than the descriptor estimation time, which is optimistically set due to the small radius of 0.01 m. The exceptions are RoPS, FPFH, and ECSAD, however with ECSAD being the only feature that also shows a high estimation efficiency. If the main focus is efficiency, we can thus conclude that the ECSAD feature is most suitable, as it provides the best overall efficiency, while showing quite high overall matching accuracy. Otherwise, RoPS and SHOT are good choices for achieving a high accuracy with a moderate efficiency, noting that for some data sources (UWA) these features show a quite serious loss of accuracy (see Fig. 7).

## The influence of the feature dimension

As shown in Estimation and matching efficiency, the total computation time for feature estimation and matching is dominated by the matching part for the high-dimensional features. Unfortunately, some of the best performing features are also those of highest dimension, which has lead us to investigate potential dimension reduction possibilities. Various methods exist for this purpose (Guyon and Elisseeff 2003), including SVD, (kernel) PCA, and spectral transforms using e.g. Fourier or wavelet series. Of the many alternatives, we opt for one of the simplest, namely PCA. We have made this choice for two reasons: 1) PCA operates on linear vector spaces, which allows for fast computation, and can thus be expected to cause limited overhead to existing feature estimation pipelines, 2) when applied to both shape and image features, the PCA subspaces have shown comparable—and in some cases even superior—performance (Johnson and Hebert 1999; Ke and Sukthankar 2004; Mikolajczyk and Schmid 2005).

For this test we use the Bologna 2 dataset. Training is performed using all features computed for the objects, i.e. no auxiliary training set is used. We take the components accounting for 99, 95, and 90 % of the variation in the training data, measured by the sum of eigenvalues in the decomposition. During runtime, the scene feature vectors are projected to the three subspaces, and we perform the rest of the analysis as in "Matching accuracy" section. We have chosen to consider the top four performing features (ECSAD, RoPS, SHOT, and USC) for clarity of presentation.

For all features except USC, the PCA training using all object features takes less than a second, and the projection of all features during runtime is very fast, taking only tens of milliseconds. For USC, which has 1980 components, the offline decomposition of the 1980 × 1980 covariance matrix and the online projection of the (on average) 4473 scene features takes tens of seconds. Although the projection time is amortized over all features, in the USC case the mean per-vertex projection time becomes higher than the estimation time. Even though the matching time is reduced by the fewer number of PCA components, the total time for estimation, projection, and matching is increased. Therefore, USC is included in this analysis for completeness, and because it is by far the feature with the highest dimension.^7^

The results of this experiment are shown in Fig. 10, with the relevant curves from Fig. 7 repeated here for direct comparison against the original features. The numbers immediately after the feature names in the legend are the dimensions of the subspace representations, accounting for the chosen variations. For RoPS, 99 % of the variation is covered by approximately half of the components, while for the other three features they cover somewhere between 95 and 99 % of the variation. For all features except ECSAD, the loss of accuracy is negligible (<1 %), even when using one third of the components. Surprisingly, we achieved a small increase in accuracy for the 99 % components for ECSAD, and for all subspace representations of USC. These results indicate that many features simply contain redundant dimensions, e.g. because the histogram bins and/or the spatial bins have been chosen too narrow. The RoPS and ECSAD features show noticeable drops in accuracy when using less than half of the components, which indicates that these features have fewer redundant components.

## Feature fusion

In this section we pick up the thread from "Matching accuracy" section and present a method for increasing the accuracy during matching. Fusion of multiple 3D features is not unknown and has been explored in other works. In Lei et al. (2013) a set of low-level 3D features are put into a histogram descriptor, which is passed to an SVM classifier to recognize faces. Daras et al. (2012) also use a number of low-level geometric features, now with the aim of retrieving 3D objects. Finally, Yang and Leng (2007) present an optimized feature selection method for combining several high-level features, again for the task of object retrieval. Common to all of these works on feature fusion is that they are able to optimize the selection of features based a supervised learning objective, i.e. by utilizing class labels from the training set. In this work, however, the task is to find good representations for matching local 3D structures in general scenes, where multiple objects and cluttering elements can occur. Put in other words, for our task there is more focus on finding a feature transform that accurately models the distribution of general local appearances, and which thereby assigns a low distance between similar structure and a high distance between dissimilar structures. In contrast, discriminative models aim at separating one class from one or more other classes. Nonetheless, we believe there are great future work prospects in using a learning algorithm to find good representations for local 3D structures.

In our fusion framework the basic idea is to combine multiple feature matches by a min pooling operation. We thus describe each seed point by multiple features, but return only one correspondence per seed point. If a seed point $\mathbf {p}$ on a query model is described by *n* feature vectors $\mathbf {f} _i(\mathbf {p}),~i=1,\ldots ,n$, we find a putative correspondence point for $\mathbf {p}$ on the target model by a nearest neighbor search in the *n* feature spaces. Denote distance ratio between the nearest target feature and the second-nearest target feature of $\mathbf {f} _i(\mathbf {p})$ as $d_{Ratio}(\mathbf {f} _i)$. Using *n* features at each point, we thus have *n* putative matches for $\mathbf {p}$, and we now take the correspondence with index *i* that minimizes the ratio distance:

$$\begin{aligned} \mathop {{{\mathrm{argmin}}}}\limits _{i} d_{Ratio}(\mathbf {f} _i) \end{aligned}$$

We initially tested this strategy with binary feature combinations ($n=2$) on the Bologna 2 dataset. For this dataset, the best performing single feature is RoPS with a $\max F_1$ score of 0.752 (see Fig. 7). As shown in Fig. 11, we get superior performance over RoPS with six out of the ten combinations tested. Two of these six combinations do not even include RoPS (ECSAD+SHOT and ECSAD+SI). It is most interesting that two of the poorly performing features on this dataset, ECSAD and SI, complement each other so well that their fusion is able to surpass the performance of RoPS.

We carried this idea further and applied the same principle with ternary feature combinations ($n=3$), leading to even better performances. The total number of possible ternary combinations is 35, but for clarity of presentation we have chosen the subset of combinations that provided the best performance, namely all possible ternary combinations of the features ECSAD, NDHist, RoPS, SHOT, and SI. The results of this experiment are shown in Fig. 12. For viewing purposes, we only included the best performing single feature along with all the ternary feature curves. Although the gain in accuracy is limited for the two Bologna 1 variants—the performances of which are already close to saturated—we achieved quite significant improvements for the remaining datasets. In particular, the increase amounted to 26 % (using ECSAD+NDHist+SHOT/SI) and 27 % (using ECSAD+RoPS+SHOT) for the object recognition datasets UWA and Queen’s, respectively. More interestingly, the fused feature matches now show a much higher level of generalization. The combination ECSAD+RoPS+SHOT consistently outperforms the best performing single feature in each dataset. The improvements of this combination over the best performing single feature for each of the datasets in Fig. 7 are as follows: 1.5, 1.4, 11, 7.8, 27, and 11 %. In other words, this feature combination produces consistently high results across all the tested datasets.

It is our belief that these results support the hypothesis that the different features cannot independently capture enough aspects of the local appearance for accurate description of different surface types. We note that the improvements here are achieved without the use of any learning algorithms, which would bias the results towards the sophistication of the learning algorithm. Indeed, by a simple operation such as min pooling, we let the best descriptor at a feature point direct the match. Clearly, this relies on the assumption that the matching distance $d _{Ratio}$ is a good indicator of a good match across different descriptors. Although the results in Fig. 12 verify this assumption, it is possible that more sophisticated pooling operations based on e.g. (learned) weighting schemes can provide even better performance.

To compare our results with an existing method, we have implemented the feature-level fusion method of Lei et al. (2013). As mentioned previously, we cannot evaluate the SVM-based score-level fusion of that work, since this would require an annotated training set, which we do not currently have. Fortunately, the feature-level fusion performed best and can be used here as a strong baseline. The results of feature-level fusion are presented in Fig. 13 and can be directly compared with the results of our method in Fig. 12. For the three Bologna datasets and for the RGB-D Scenes, the feature-level fusion actually performs weaker than both the original single features as well as our fused matches. However, for the UWA and Queen’s datasets, feature-level fusion clearly works better than single features. For UWA, the feature-level combination NDHist+RoPS+SI achieves a $\max F_1$ score of 0.394, and for Queen’s the same combination achieves a $\max F_1$ score of 0.129. In all datasets, however, our fusion method produces superior results. For the two first Bologna datasets, which are less challenging, our fusion method shows marginal improvements, whereas for the other four datasets the improvements are quite substantial.

In the following section, we present a final application of the various optimizations presented in this work, arguing for the advantage of using feature fusion during object recognition.

## 3D object recognition benchmarks

As a final contribution of this work, we present benchmark results for all our single and ternary features using the two object recognition datasets, UWA and Queen’s. To this end, we employ a simplistic, RANSAC-based pose estimation algorithm for recognizing objects in a scene. The full processing pipeline for an input scene can be summarized by the diagram in Fig. 14, which is a specialization of the general structure in Fig. 1. In the following paragraphs we shortly describe the individual steps.

### Seed points and feature descriptors

We use the same uniform object seed points as in the accuracy tests in "Matching accuracy" section, but for the scenes we cannot exploit the ground truth pose for selecting exact matches. Therefore we double the resolution of the target seeds to get a better chance of describing the same feature points as in the object models. The scenes are all 2D manifolds, so double the seed resolution approximately quadruples the number of feature descriptors.

For describing objects and scenes, we use the PCA-reduced features presented in "The influence of the feature dimension" section, the number of components set so they cover 99 % of the variation in the data. Feature description, PCA training and projection of the object models is all performed offline, while feature description and PCA projection of the more densely covered scene model is done online. Similar to "Feature fusion" section, we consider only the top performing subset of features for our tests.

### Matching and fusion

Even though the PCA reduction in the previous paragraph allows for higher matching efficiency, we now apply approximate techniques for finding nearest and second-nearest matches. To this end, we use the FLANN (Muja and Lowe 2014) library, which allows for orders of magnitudes of speedup when searching high-dimensional feature spaces. This is achieved by multiple randomized *k*-d trees, with a bound on the number of checks to perform while searching. We use four trees and a bound of 512 checks for a good trade-off between accuracy and efficiency. All matches are ranked by the $L_2$ distance ratio, providing putative input correspondences to the RANSAC algorithm.

In Fig. 14, an optional fusion step is included, which implements the method presented in "Feature fusion" section. For our recognition benchmarks we test single features (no fusion) and ternary features. Unlike all other processes, we have CPU parallelized the fusion process from the feature description stage, over the matching step, to the actual fusion of ternary matches using min pooling. We have chosen to do so, since at each of these stages the operations are completely independent of each other. Additionally, multi-core CPU architectures are ubiquitous, which makes the fusion method easily applicable.

### Pose estimation + refinement and segmentation

The input to RANSAC, the *data points*, are in the case of pose estimation the computed feature correspondences, either using single feature matches or fused matches. Each of these correspondences has an associated quality score given by the $L_2$ distance ratio. During random sampling, three correspondences are sampled, which is enough for generating a hypothesis pose, which is then verified by the number of supporting data points. For a better test of the performance of different features, our pose estimation algorithm deviates from classical RANSAC (Fischler and Bolles 1981) in the way we sample correspondences. Unlike classical RANSAC, which treats all data points uniformly, we sample each correspondence with a probability equal to its quality. We define the quality of the *i*'th correspondence as the negative of the matching distance, normalized to produce a distribution:

$$\begin{aligned} q_i = \frac{ -d _{Ratio,i} }{ \sum -d _{Ratio} } \end{aligned}$$

Furthermore, to filter out the least promising matches, we discard the 50 % correspondences of lowest quality before executing RANSAC. For verification of a sampled pose, we now count supporting data points or *inliers*. In contrast with other works which often use full object models (Guo et al. 2013; Mian et al. 2006), sometimes with sophisticated penalty functions (Aldoma et al. 2012; Papazov and Burschka 2011), our method of verification applies that of classical RANSAC. In other words, when a hypothesis pose has been generated, we apply the pose to each query feature point in the pool of input correspondences, and we count how many of the transformed feature points lie close to their corresponding target feature point up to a tolerance given by the seed point resolution. The algorithm returns the pose with highest inlier count. To filter out false positives, we set a lower limit for the inliers of 5 % of the number of input correspondences. The objects are processed by RANSAC in order of decreasing number of correspondences in the scene.

Upon completion of RANSAC, the output pose, if any, is refined using ICP (Besl and McKay 1992) on the query/target seed points. Finally, the object is aligned with the corresponding scene data using the refined pose, and the part of the scene occupied by the object is segmented out before moving on to the next query object.

The RANSAC and ICP loops are run for 1000 and 50 iterations, respectively, per object. The fact that we use the precomputed feature point matches during RANSAC makes the algorithm very fast, and the recognition rate is expected to be proportional to the quality of these matches. Thus, a good choice of features is expected to allow for both efficient and accurate recognition. To determine if an object is correctly recognized, we compare the output pose with the ground truth pose provided by the dataset. Denote the ground truth rotation and translation as $\mathbf {R}$ and $\mathbf {t}$, respectively, and the estimated rotation/translation by $\hat{\mathbf {R}}$ and $\hat{\mathbf {t}}$. A true positive is determined by imposing an upper bound on the rotation/translation errors, with the rotation error computed using the geodesic distance on *SO*(3):

$$\begin{aligned}&\arccos \left( \frac{{{\mathrm{trace}}}\left( \mathbf {R}^T\hat{\mathbf {R}}\right) - 1}{2} \right) \le 7.5^\circ \nonumber \\&\Vert \mathbf {t} - \hat{\mathbf {t}}\Vert \le 0.05~\text {m} \nonumber \end{aligned}$$

The translation error bound is set fairly high, as we noticed that quite a number of the ground truth poses were fairly inaccurate, leading to a excessive number of false negatives under more conservative error bounds.

### Recognition results, UWA

The full set of recognition results for the UWA dataset can be seen in Table 4. For comparison, we have included state of the art results for external systems such as Spin Images (Johnson and Hebert 1999), the original work using Tensor Matching (Mian et al. 2006), Point Pair Features (PPF) with two discretization levels of the PPF feature space (Drost et al. 2010), Exponential Maps (EM) (Bariya et al. 2012), and finally the RoPS based system (Guo et al. 2013). The accuracy is shown as recognition rates (equal to recall), while efficiency is listed as the average per-object time required for detecting a single object in a scene. Regarding the timings, it should be noted that the Spin Images and Tensor Matching systems are based on MATLAB implementations, while the rest are based on C++ implementations. For RoPS (also implemented in MATLAB), timing information is not available, but it is expected to be at least as high as for the Queen’s dataset, which is the case for all other systems (see Table 6).

**Table 4:** Recognition rates and and per-object detection times (where available) for the UWA scenes

Method	Recognition rate	Timing (s)
External systems		
Spin Images	0.878	$\sim$7200
Tensor Matching	0.966	$\sim$90
PPF, $\tau _d=0.025$	0.970	85
PPF, $\tau _d=0.04$	0.892	*1.97*
EM	0.975	–
RoPS based	*0.989*	–
RANSAC, single feature		
ECSAD	0.947	1.139
NDHist	*0.968*	1.155
RoPS	0.846	1.254
SHOT	0.809	1.221
SI	0.803	*0.998*
RANSAC, ternary feature		
ECSAD+NDHist+RoPS	0.979	1.253
ECSAD+NDHist+SHOT	0.979	1.192
ECSAD+NDHist+SI	0.968	1.116
ECSAD+RoPS+SHOT	0.947	1.187
ECSAD+RoPS+SI	0.957	*1.095*
ECSAD+SHOT+SI	0.957	1.103
NDHist+RoPS+SHOT	0.952	1.182
NDHist+RoPS+SI	0.957	1.102
NDHist+SHOT+SI	*0.984*	1.112
RoPS+SHOT+SI	0.931	1.227

Italics highlight the best (lowest) recognition times for each group of systems

We immediately observe the high recognition rates of most of our systems, and the general improvement achieved by using ternary features. Almost all ternary combinations show state of the art performance, which again demonstrates the ability of the different features to complement each other.

These results are only intended for analysis purposes, but the unexpectedly high recognition rates led us to include the external results for state of the art, complex recognition systems. We stress that our results are not to be taken as a argumentation for the use of our presented recognition algorithm over existing systems, due to the single fact that our features use support radii which have been tuned for optimal performance. Comparisons with external systems are merely included for clarity, and for a good indication of what is achievable. That being said, we find it quite interesting that our simplistic algorithms, especially using ternary features, are able to compete with—and in some cases even surpass—the recognition performance of recent systems. Due to the relative simplicity of our systems, we achieve our results in significantly shorter time than virtually all other systems, the best competitor being the coarse PPF algorithm.

The timings of our single feature systems are further detailed in Table 5. Note that the resources spent on preprocessing the input scene (decimation and seeds) is amortized over all four objects tested. From these timings it can be observed that the initial decimation of the scene takes up approximately 30 % of the total processing time. The matching time is brought significantly down, partially by the use of FLANN, but also by the PCA representation. The online PCA projection is a very fast operation for all the included features, and amounts to only 3 % of the processing time.

**Table 5:** Decomposition of the total per-object detection timings of each of the single feature systems for the UWA dataset

Feature	Decimation	Seeds	Feature estimation	PCA projection	Matching	RANSAC	ICP	Segmentation
ECSAD	0.307	0.002	0.013	0.000	0.053	0.549	0.211	0.003
NDHist	0.307	0.002	0.019	0.005	0.069	0.537	0.213	0.003
RoPS	0.307	0.002	0.149	0.005	0.065	0.520	0.202	0.003
SHOT	0.307	0.002	0.050	0.036	0.079	0.536	0.208	0.003
SI	0.307	0.002	0.006	0.007	0.067	0.440	0.166	0.002

### Recognition results, Queen’s

Recognition results for the Queen’s dataset are presented in Table 6, including results for the original work on Variable-Dimensional Local Shape Descriptors (VD-LSD) (Taati and Greenspan 2011), EM, and the RoPS based system. The timing for VD-LSD is provided only for one object, *BigBird*. This model is the second-smallest in the object set, making this estimate slightly optimistic.

**Table 6:** Recognition rates and and per-object detection times (where available) for the Queen’s scenes

Method	Recognition rate	Timing (s)
External systems		
VD-LSD (SQ)	0.838	*2.964*
EM	0.824	–
RoPS based	*0.954*	153.8
RANSAC, single feature		
ECSAD	0.791	0.659
NDHist	0.430	*0.495*
RoPS	0.676	0.766
SHOT	*0.841*	0.742
SI	0.618	0.565
RANSAC, ternary feature		
ECSAD+NDHist+RoPS	0.845	0.788
ECSAD+NDHist+SHOT	0.826	0.719
ECSAD+NDHist+SI	0.836	0.632
ECSAD+RoPS+SHOT	0.894	0.753
ECSAD+RoPS+SI	0.894	0.698
ECSAD+SHOT+SI	*0.913*	0.679
NDHist+RoPS+SHOT	0.836	0.674
NDHist+RoPS+SI	0.802	0.627
NDHist+SHOT+SI	0.826	*0.626*
RoPS+SHOT+SI	0.879	0.797

Italics highlight the best (lowest) recognition times for each group of systems

The recognition results for Queen’s follow the tendency from UWA, however being slightly more difficult, but also faster due to the sparser object/scene models. For this dataset, the speedup is even more pronounced. More interestingly, the best ternary combination ECSAD+SHOT+SI now performs significantly better than the top single feature performer, giving a notable increase in accuracy with a limited penalty on efficiency. The precision values of the top performers SHOT and ECSAD+SHOT+SI are 0.798 and 0.818, respectively.

In general, the performances of our single features correlate well with the accuracy results. Indeed, the top performer for UWA matching accuracy, NDHist, is also a clear top performer during recognition. Similarly, SHOT together with ECSAD showed the highest accuracy for Queen’s, and these features also outperform the other features by a large margin in terms of recognition rate. The high performances of SI and RoPS based on external systems are therefore attributable solely to the recognition systems in these works, which are hundreds times slower than our RANSAC algorithm.

### Qualitative recognition results

We end this presentation with qualitative recognition results in Fig. 15. For the UWA dataset, we show a scene (top row) where three features fail by producing misalignments (false positives). Their ternary combination, however, picks a better set of matches for the feature points, which allows RANSAC to produce the correct pose maximizing the inlier count. The next two rows show a scene from the Queen’s dataset. For this scene, the single features in the middle row produce both false positives (a *Gnome* in the second row of Fig. 15) and a generally high amount of false negatives (also in the same part of Fig. 15 since the *Angel* is not detected although it is present). Nine out of ten ternary combinations perform at full recognition rate and precision in this scene, as shown in the bottom row.

## Conclusion

We have presented a thorough analysis covering several aspects of local 3D shape descriptors, such as matching accuracy, estimation and matching efficiency, dimension reduction, feature fusion and object recognition.

We have shown that many of the recently proposed feature descriptors, which provide very high matching accuracy for synthetic data, do not perform well when exposed to the real data with disturbances (e.g. occlusions) used in our experiments. Our fused features overcome this problem and show top performances over all tested datasets. Although our experimental data covers a wide range of different scenarios and shape variations, the results cannot be seen as universal. It is an interesting extension of this work to include even more external datasets, e.g. from other sensor types.

Using our fused features, we are able to match and in some cases supersede the performance of several recent methods for 3D pose estimation and object recognition. To reduce the processing time during feature matching, we also showed how a PCA reduction can be performed, causing an insignificant decrease in matching accuracy. All in all, our methods achieve recognition rates of 96.8 and 91.3 % for the UWA and Queen’s datasets, respectively, in only 1 s of processing time per object. Similar recognition rates are achieved in one or two orders of magnitude longer time by state of the art 3D object recognition pipelines.

For future works, we believe more research is required for arriving at features with better generalization properties. This can be achieved either by further exploring feature fusion as in this work, or by other means. One important research topic should be to bring the recent works on deep architectures to the 3D domain, since such features have shown a high degree of generalization for various matching tasks. In line of this research, we believe that for a system to be widely applicable, a *feature zoo* of many different features may be necessary. In this work, we have fused different shape descriptors, but in general it should be possible to combine features capturing several aspects of an object, e.g. shape and appearance, local and (semi-)global cues, etc. Furthermore, for true generalization, subsets of features should be automatically selected based on the nature of the input data, be it from synthetic sources, real sensors, or something completely different. Future solutions to this problem could be based on the recent developments on artificial neural networks (Gupta et al. 2014; Hinton et al. 2006; Krizhevsky et al. 2012), by which (semi-)global representations are learned, partially in an unsupervised manner. It is an open question whether such representations are suitable for describing local 3D shape information.

## Footnotes

1 Not to be confused with the specificity of classification systems, where this term refers to the true negative rate.

2 Indeed, most features are $L_1$ normalized to produce a relative histogram. The manifold of these feature vectors is thus a high-dimensional diamond shape. The SHOT feature vectors are $L_2$ normalized, and thus lie on a unit hypersphere.

3 http://www.pointclouds.org.

4 https://www.gitlab.com/caro-sdu/covis.

5 http://www.graphics.stanford.edu/data/3Dscanrep.

6 http://www.meshlab.sourceforge.net.

7 The specific timings mentioned here are dependent on processor architecture, but the argument holds in general since both PCA decomposition and projection are superlinear operations in the feature dimension.
